# Controversies regarding lithium-associated weight gain: case–control study of real-world drug safety data

**DOI:** 10.1186/s40345-023-00313-8

**Published:** 2023-10-15

**Authors:** Waldemar Greil, Mateo de Bardeci, Bruno Müller-Oerlinghausen, Nadja Nievergelt, Hans Stassen, Gregor Hasler, Andreas Erfurth, Katja Cattapan, Eckart Rüther, Johanna Seifert, Sermin Toto, Stefan Bleich, Georgios Schoretsanitis

**Affiliations:** 1 Department of Psychiatry and Psychotherapy, Ludwig Maximilian University, Nussbaumstr. 7, 80331 Munich, Germany; 2 Psychiatric Private Hospital, Sanatorium Kilchberg, Zurich, Switzerland; 3 Department of Psychiatry, Psychotherapy and Psychosomatics, Hospital of Psychiatry, University of Zurich, Zurich, Switzerland; 4 Charité Universitätsmedizin-Berlin, Berlin, Germany; 5 Medical Faculty Brandenburg Theodor Fontane, Neuruppin, Germany; 6 Drug Commission of the German Medical Association, Berlin, Germany; 7 IFMA Preventive Health Management Inc., 80 Pine Street, 24th Floor, New York, NY 10005 USA; 8 Psychiatry Research Unit, University of Fribourg, Fribourg, Switzerland; 9 Klinik Hietzing, 1st Department of Psychiatry and Psychotherapeutic Medicine, Vienna, Austria; 10 University Hospital of Psychiatry and Psychotherapy, University of Bern, Bern, Switzerland; 11 Department of Psychiatry, Social Psychiatry and Psychotherapy, Hannover Medical School, Hannover, Germany; 12 The Zucker Hillside Hospital, Psychiatry Research, Northwell Health, Glen Oaks, NY USA; 13 Department of Psychiatry at the Donald and Barbara Zucker School of Medicine at Northwell/Hofstra, Hempstead, NY USA

**Keywords:** Weight gain, Lithium, Mood stabilizer, Adverse drug reaction (ADR), Case–control study, Drug safety, Pharmacovigilance

## Abstract

**Background:**

The impact of long-term lithium treatment on weight gain has been a controversial topic with conflicting evidence. We aim to assess reporting of weight gain associated with lithium and other mood stabilizers compared to lamotrigine which is considered free of metabolic adverse drug reactions (ADRs).

**Methods:**

We conducted a case/non-case pharmacovigilance study using data from the AMSP project (German: “Arzneimittelsicherheit in der Psychiatrie”; i.e., Drug Safety in Psychiatry), which collects data on ADRs from patients treated in psychiatric hospitals in Germany, Austria, and Switzerland. We performed a disproportionality analysis of reports of weight gain (> 10% of baseline body weight) calculating reporting odds ratio (ROR). We compared aripiprazole, carbamazepine, lithium, olanzapine, quetiapine, risperidone, and valproate to lamotrigine. Additional analyses related to different mood stabilizers as reference medication were performed. We also assessed sex and age distributions of weight-gain reports.

**Results:**

We identified a total of 527 cases of severe drug-induced weight gain representing 7.4% of all severe ADRs. The ROR for lithium was 2.1 (95%CI 0.9–5.1, p > 0.05), which did not reach statistical significance. Statistically significant disproportionate reporting of weight gain was reported for olanzapine (ROR: 11.5, 95%CI 4.7–28.3, p < 0.001), quetiapine (ROR: 3.4, 95%CI 1.3–8.4, p < 0.01), and valproate (ROR: 2.4, 95%CI 1.1–5.0, p = 0.03) compared to lamotrigine. Severe weight gain was more prevalent in non-elderly (< 65 years) than in elderly patients, with an ROR of 7.6 (p < 0.01) in those treated with lithium, and an ROR of 14.7 (p < 0.01) in those not treated with lithium.

**Conclusions:**

Our findings suggest that lithium is associated with more reports of severe weight gain than lamotrigine, although this difference did not reach statistical significance. However, lithium use led to fewer reports of severe weight gain than some alternative drugs for long-term medication (olanzapine, quetiapine, and valproate), which is consistent with recent studies. Monitoring of weight gain and metabolic parameters remains essential with lithium and its alternatives.

**Supplementary Information:**

The online version contains supplementary material available at 10.1186/s40345-023-00313-8.

## Background

The impact of lithium on weight gain has been a topic of ongoing debate. Previous studies have suggested that treatment with lithium may lead to significant weight gain, with approximately one-third of lithium users experiencing this side effect (e.g., Swiss package insert: https://compendium.ch/search?q=lithium) (Gitlin 2016), which in turn has been reported among leading reasons for lithium discontinuation (Ohlund et al. 2018; Rybakowski and Suwalska 2013). However, a recent meta-analysis challenged this notion (Gomes-da-Costa et al. 2022), as they suggested that lithium treatment does not cause substantial weight gain compared to other psychotropic drugs such as lamotrigine, venlafaxine, valproate, and various second-generation antipsychotics (SGAs), especially risperidone, quetiapine, and olanzapine. This finding contradicts previously established beliefs about the effects of lithium on body weight and highlights the need for further research in this area. Additionally, it has been suggested that the cause of weight gain in individuals taking lithium may be falsely attributed to lithium use, as it may be due to the underlying affective disorder itself rather than the drug; in fact, patients with bipolar disorders are at increased risk for obesity and metabolic abnormalities regardless of the effects of the pharmacological treatment (Reilly-Harrington et al. 2018). Specifically, large-scale epidemiological data reported an increased frequency of 1.5 for obesity in patients with bipolar disorders compared to patients without (41.% versus 27%, Sicras et al. 2008). Additionally, some of the observed metabolic effects may be confounded by the fact that patients frequently suffer from baseline obesity before starting treatment with lithium (Kemp et al. 2014; McElroy et al. 2016). Some underline the predictive role of obesity regarding treatment outcomes, with obese patients being at high risk for relapse in the long term (Fagiolini et al. 2003). Obese patients with bipolar disorders may present a patient subgroup with more severe illness manifestations as they may suffer from more severe symptoms of longer duration (Fagiolini et al. 2003); a potential confounder for this pattern may be the role of adherence to pharmacological treatment, which may be negatively associated with weight gain related to the prescribed medication (Youn et al. 2022). In other words, bipolar patients suffering from weight gain related to lithium or other mood stabilizers are less likely to be adherent (Youn et al. 2022) and, thus, more likely to relapse (Bauer et al. 2019). Further, the use of multiple drugs (i.e., polypharmacy) which includes lithium, may also cause adverse metabolic reactions (Gomes-da-Costa et al. 2022). Therefore, it is important to understand the various factors underlying metabolic abnormalities in patients with bipolar disorders and particularly the role of the short-, middle-, and long-term treatment with mood stabilizers. According to most national and international guidelines, lithium is considered the first-choice long-term episode-preventing strategy, whereas anticonvulsants, such as valproate, lamotrigine, and carbamazepine also have an established role (Fountoulakis et al. 2017). Pharmacoepidemiological data worldwide point out that SGAs are increasingly being prescribed for the maintenance treatment of bipolar disorders (Bartoli 2023). Also, polypharmacy is routine practice ranging from 36% up to 85% of patients in real-world hospital settings (Fornaro et al. 2016). When choosing between mood stabilizers, the safety profile of these drugs should be given careful consideration with special regard to the risk of weight gain and/or metabolic abnormalities. Robust data on weight gain associated with mood stabilizers are now available from more recent randomized clinical trials (Grootens et al. 2018; Schoretsanitis et al. 2018). They show that some drugs (e.g., olanzapine) are known for their particularly high risk of metabolic abnormalities (Huhn et al. 2019). However, the evidence for the corresponding risks of other mood stabilizers is less robust and sometimes controversial (Yatham et al. 2018).

Pharmacovigilance data have significant value in this context. They capture variations in safety profile that are seen in real-world clinical practice rather than in randomised clinical trials (RCTs). RCTs tend to focus on monotherapy or adjunctive treatments for moderately severe conditions with no comorbidities, rather than on complex polypharmacy for severe conditions often associated with comorbidities. The results of pharmacovigilance data are therefore a key factor in assessing the metabolic profile of mood stabilisers in both the short and, importantly, the long term.

We aim to assess the risk of weight gain associated with lithium compared to other mood stabilizers in a large pharmacovigilance dataset reflecting real-world clinical conditions. It complements a previous publication on weight gain with psychotropic drugs in general, based on data from the same pharmacovigilance program (Schneider et al. 2020).

## Methods

### Data sources

The AMSP project (German: “Arzneimittelsicherheit in der Psychiatrie”; i.e., Drug Safety in Psychiatry) is a pharmacovigilance program that was established in 1993 and collects real-world data on adverse drug reactions (ADRs) in patients from psychiatric hospitals in Germany, Austria, and Switzerland (Schneider et al. 2020). In the present study, we analyzed data from 60 university, municipal and state psychiatric hospitals and departments that participated in the AMSP program during the time period from 2001 to 2015*.* In this study, we focused on the dataset containing reports of severe ADRs. In a cross-sectional approach, the participating hospitals record severe ADRs using the AMSP protocol that provides specific standardized guidelines for the assessment of ADRs. Generally, ADRs are regarded as “severe” when causing a substantial impact on the course of treatment (e.g., life-threatening or seriously endangering health) or on the patient's everyday functioning.

### Definition of severe weight gain

We report cases of severe weight gain (SWG) that were potentially associated with one or more psychotropic drugs. Within the AMSP consensus, SWG was defined as an increase in weight of more than 10% of the baseline body weight since 2001 (Grohmann et al. 2014). Weight gain that compensated for prior weight loss due to psychiatric symptoms is not taken into account. This was because the definition of SWG did not consider this aspect.

From 1993 (i.e., when AMSP was founded) until 2000, SWG included only cases where the Body Mass Index (BMI) increased above 30 kg/m^2^ (Grohmann et al. 2004). Consequently, for the present study, we only considered data collected from 2001 onwards. Additionally, this study took into account the presence of weight gain-related conditions noted in the patient’s medical records, specifically weight gain accompanied by hyperlipidemia and weight gain associated with metabolic syndrome.

### Study population

From the dataset comprising a total of 9593 cases of (mostly) severe ADRs between 1993 and 2015, all cases with psychiatric diagnoses according to the International Classification of Diseases, 10th Revision (ICD-10), documented between 2001 and 2015 and having severe ADRs (all types of ADRs) were included. ADRs that led to hospitalization or discontinuation of medication were also included. A total of 7104 cases of ADRs were included for analysis.

### Data analysis

To assess the relationship between the incidence of weight gain as an ADR with the prescription of various psychotropic drugs, we performed a comparative case–control analysis employing reported odds ratios (RORs). This case/non-case design is well established and has been used in previous analyses using the AMSP dataset (Greil et al. 2019). For each drug, we computed the RORs using a 2 × 2 contingency table, which included the following values: instances of reference drug A without drug B, instances of drug B without reference drug A, cases of SWG and cases without SWG. By examining the data through these contingency tables, we determined the RORs, their corresponding 95% confidence intervals (CIs), and p-values for each drug (χ^2^-test). For the analysis, we used software developed in R (version 4.2.2).

Our analysis included the following drugs (in alphabetical order): aripiprazole, carbamazepine, lamotrigine, lithium, olanzapine, quetiapine, risperidone, and valproate. Lamotrigine was used as the reference drug for the primary outcome comparisons since it is not known to cause significant weight gain (Grootens et al. 2018). Additional analyses were performed using other drugs as reference: lithium, carbamazepine, and aripiprazole, as well as the group of high-potency first-generation antipsychotics. In this analysis, we took all psychotropic drugs prescribed to a patient into critical consideration, regardless of causality assessments by the responsible clinicians to avoid possible physician bias. Data are kept anonymously to prevent the identification of subjects*.* Assessments based on the AMSP database have been approved by the Ethics Committee of the University of Munich and the Ethics Committee of the Hannover Medical School (Nr. 8100_BO_S_2018). This study adheres to the Declaration of Helsinki and its later amendments. The AMSP program is a continuous observational post-marketing drug surveillance program and does not interfere with the ongoing clinical treatment of the patients under surveillance.

## Results

Totally, 527 cases of severe drug-induced weight gain (> 10% of body weight) were collected, representing 7.4% of all ADRs (527 out of 7104 cases).

### Weight gain and sex and age differences

SWG was found in 7.9% of ADRs in women and in 6.8% of ADRs in men (p > 0.05), i.e. there was no statistically significant sex difference for SWG in comparison to all ADRs. SWG was more common in patients younger than 65 years than in patients ≥ 65 years. This was true for both the lithium group and the patients receiving other non-lithium psychotropic drugs. In this comparison of age groups, the ROR was 7.6 for lithium (p < 0.01) and 14.7 for non-lithium (p < 0.01). The corresponding values for the comparison < 45 years and ≥ 45 years were as follows: Lithium ROR 11.0; p < 0.01, non-lithium: ROR 3.9, p < 0.01. In summary, there was a distinctly higher relative incidence of SWG in the younger age group < 45 years for lithium compared to non-lithium drugs (ROR 11.0 vs. 3.9).

### Weight gain and medication

**Fig. 1:**
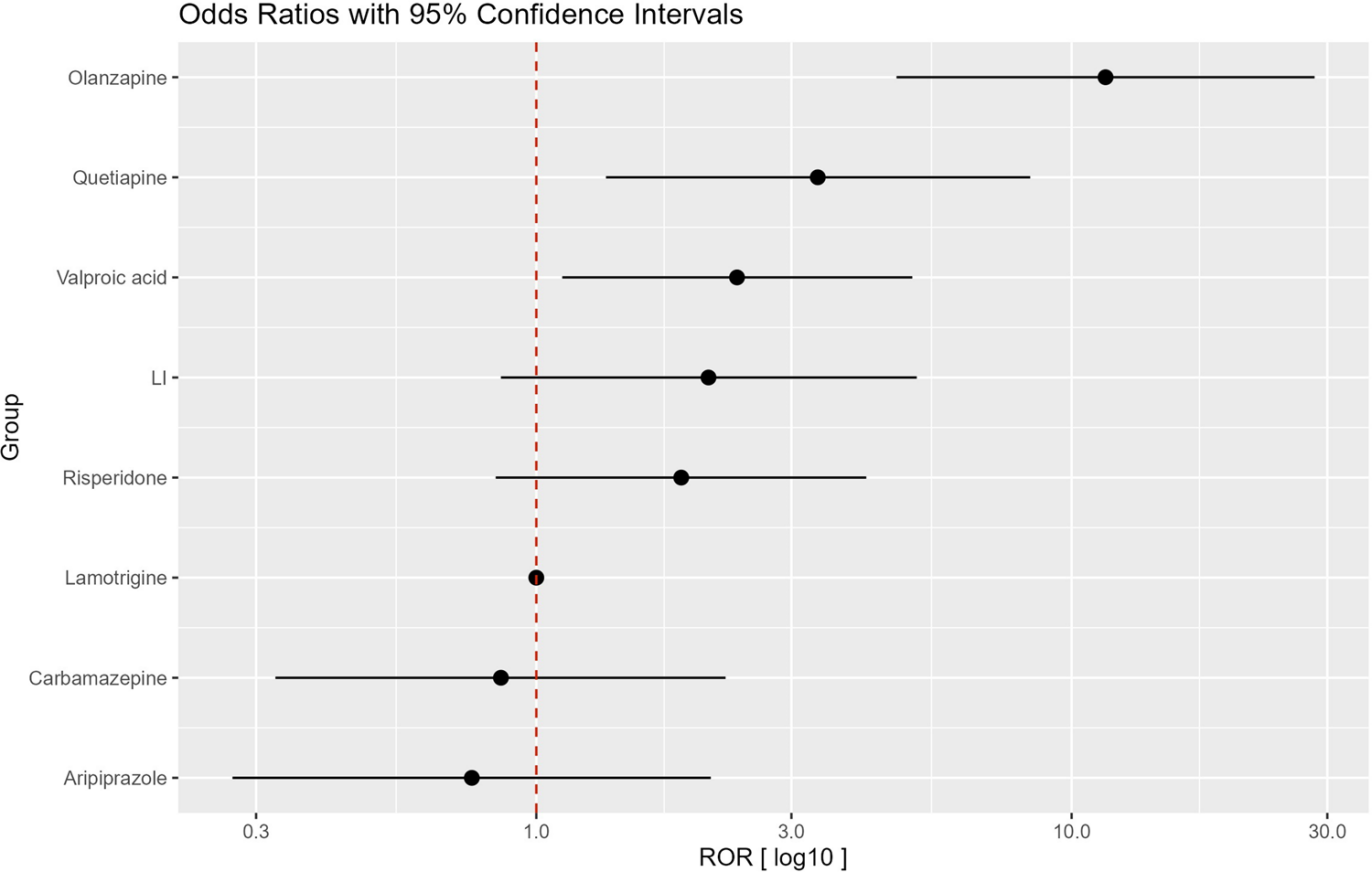
Reporting odds ratios (RORs) and 95% confidence intervals (95%CI) of cases (N: 527) of severe weight gain (> 10% of baseline) between 2001 and 2015 for aripiprazole, carbamazepine, risperidone, lithium (LI), valproate, quetiapine, and olanzapine compared to lamotrigine

We found a statistically significant disproportionate reporting of SWG, i.e., higher association for olanzapine (ROR: 11.5, 95%CI 4.7–28.3, p < 0.001), quetiapine (ROR: 3.4, 95%CI 1.3–8.4, p < 0.01), and valproate (ROR: 2.4, 95%CI 1.1–5.0, p = 0.03) compared to lamotrigine (Table 1, Fig. 1).

**Table 1:** Reporting odds ratios (RORs) and 95% confidence intervals (95%CI) of severe weight gain for the drugs investigated (in brackets number of cases)

Medication (n)	ROR (95%-CI)	p-value (χ^2^ test)
Aripiprazole (9)	0.8 (0.3–2.1)	pval = 0.8
Carbamazepine (9)	0.9 (0.3–2.3)	pval = 0.95
Lamotrigine (8)	1	
Risperidone (60)	1.9 (0.8–4.1)	pval = 0.2
Lithium (31)	2.1 (0.9–5.1)	pval = 0.14
Valproate (61)	2.4 (1.1–5.0)*	pval = 0.03
Quetiapine (104)	3.4 (1.3–8.4)*	pval = 0.009
Olanzapine (247)	11.5 (4.7–28.3)*	pval = 0.0005

*statistically significant (p<0.05)

Furthermore, the differences in the reporting of SWG for lamotrigine in comparison to lithium (ROR = 2.1, 95%CI 0.9–5.1) and risperidone (ROR 1.9, 95%CI 0.8–4.1) were substantial but did not reach the significance level of 5%. Additionally, the differences in comparison to carbamazepine (ROR: 0.9, 95%CI 0.3–2.3) and aripiprazole (ROR 0.8, 95%CI 0.3–2.1) were very small and not statistically significant.

Thus, the hierarchical ranking order of drugs for the occurrence of SWG from highest to lowest was olanzapine, quetiapine, valproate, lithium, risperidone, lamotrigine, carbamazepine, and aripiprazole. The order remained unchanged when lithium, carbamazepine, or aripiprazole was chosen as the reference drug (for details see Additional file 1: Figure S1–3).

In a further step, we compared SWG reporting related to lithium with different groups of psychotropic drugs. We did not find statistical differences between lithium and selective serotonin reuptake inhibitors (SSRIs), selective serotonin-norepinephrine reuptake inhibitors (SNRIs), noradrenergic and specific serotonergic antidepressants (NaSSA), tricyclic antidepressants (TCAs), and antiepileptic drugs (AEDs). Consistent with our finding above, we noted increased SWG reporting in patients treated with SGA compared to patients treated with lithium (ROR 4.0, p < 0.01), whereas high-potency first-generation antipsychotics (FGA) had significantly less reported SWG (ROR 0.6, p < 0.05, see Additional file 1: Figure S5).

## Discussion

In our analysis of pharmacovigilance data using lamotrigine as reference drug, olanzapine, quetiapine, and valproate had significantly higher RORs for weight gain compared to other drugs, which are frequently used in long-term treatment of patients with bipolar disorder: lithium, lamotrigine, and aripiprazole. These findings are strikingly consistent with meta-analytic data from RCTs conducted in patients with bipolar mania (Kishi et al. 2022, see also supplement, page 313). In this network meta-analysis, authors reported a higher risk of weight gain for olanzapine, quetiapine, asenapine, ziprasidone, paliperidone, and ziprasidone compared to placebo, whereas aripiprazole, carbamazepine, lithium, and risperidone were void of such side effects (Kishi et al. 2022). Comparison of RORs in our case–control study (related to lamotrigine) and in the meta-analysis by Kishi et al. (related to placebo) show good agreement: ROR for lithium 2.1 versus 1.9, risperidone 1.9 versus 2.1, for quetiapine 3.4 versus 4.1, and for olanzapine 11.5 versus 8.2. This indicates a high validity of our evaluation, including the results on lithium. The deleterious metabolic profile of olanzapine has been well-established in studies of varying designs (Huhn et al. 2019; Schoretsanitis et al. 2022), and there have been efforts to mitigate this ADR by a special drug combination, such as combining olanzapine with samidorphan (Meyer et al., 2022). A previous analysis of the AMSP dataset also suggested an average weight gain of 1.8 kg/week in patients treated with olanzapine (Schneider et al. 2020).

The association of quetiapine with weight gain is also well-known, with novel data warning that even low-dose quetiapine is associated with this adverse effect (Hojlund et al. 2023). This appears particularly relevant as low-dose quetiapine is frequently prescribed as an add-on drug targeting secondary symptoms such as insomnia and agitation in a transdiagnostic fashion (de Bardeci et al. 2023; Pringsheim and Gardner 2014). In the present AMSP study, SWG associated with quetiapine was reported 3.4 times more frequently compared to lamotrigine-associated weight gain, which was less severe than for olanzapine.

Regarding the metabolic profile of valproate, previous data from RCTs, but also from naturalistic settings, consistently suggest a risk for weight gain (Bowden et al. 2010; Zuo et al. 2015). Specifically, a 12-weeks study in which randomized patients with mania or mixed mania to treatment with valproate or lithium reported more weight gain in patients prescribed valproate than in those treated with lithium [average weight gain: 1.1 kg versus 0.2 kg over the trial period (Bowden et al. 2010)].

Of particular interest is the increased body weight in patients treated with lithium. Although our data suggest a twofold more frequent reporting of weight gain associated with lithium compared to lamotrigine, this result was not statistically significant. Evidence from randomized clinical trials also suggests a low risk of weight gain in patients treated with lithium (Gomes-da-Costa et al. 2022; Kishi et al. 2022). No statistically significant or substantial associations between lithium use and body weight have been described in various studies (Bopp et al. 2019; Bowden et al. 2006; Findling et al. 2019; Phelps et al. 2021; Prillo et al. 2021; Pruccoli et al. 2022; Solmi et al. 2020; Yaramala et al. 2020). For example: Among 583 elderly patients with bipolar disorder, those treated with lithium (n = 182) had a statistically significant lower body weight than those not treated with lithium (mean: 79 kg versus 85 kg; Forlenza et al., 2022). Findings from a randomized study over the period of 2.5 years with 74 patients with bipolar disorder suggest weight gain (combined with acne) results in dropout from the trial in only a single case (Greil et al. 1997). The comparatively favorable profile of lithium with respect to SWG compared to olanzapine and to SGAs (as a group) was also suggested in a previous AMSP study using a different evaluation method (taking into account the imputations of the individual drugs and calculating the relation of SWG to the prescriptions of the drugs) (Schneider et al. 2020). As to the predictability of this ADR, it appears of practical importance that obese or overweight patients may be at higher risk of weight gain associated with lithium compared to patients with normal baseline body weight (Rybakowski and Suwalska 2006; Bowden et al. 2006; Greil 1981). In non-obese patients—as opposed to patients with pre-existing obesity—almost no weight gain was seen with lithium compared with placebo and lamotrigine. At week 52, weight gain was − 0.5, + 1.1, and + 0.7 kg with lamotrigine, lithium, and placebo, respectively, with no significant differences between the groups (Bowden et al. 2006). Serum lithium levels have historically been maintained at higher levels (0.8–1.2 mmol/l) than today (0.6–0.8 mmol/l or even lower), particularly in the United States (Bauer et al. 2013) and the weight-increasing effect of lithium may be serum level-dependent (Gelenberg et al. 1989). Consequently, the nowadays comparatively lower serum lithium levels could be responsible for the more favorable results in recent studies.

The age dependency of SWG was already shown in a previous analysis of AMSP data: higher age was associated with a lower incidence of SWG, especially for olanzapine (Greil et al. 2013). This effect is also observed in the present study for lithium-associated SWG, especially when comparing patients who are younger than 45 to those who are 45 and older.

Even if the weight-increasing side effect of lithium appears to be smaller than previously assumed, it remains essential to inform patients about the risk of weight gain when starting treatment with lithium. Further, a detailed monitoring of weight and metabolic parameters before and during drug treatment may help clinicians to disentangle lithium effects from pre-existing co-morbidities, such as baseline obesity (Kemp et al. 2014; McElroy et al. 2016). Body weight has to be assessed regularly and frequently by patients and during each visit to their psychiatrists or lithium clinics (Felber et al. 2018; Youn et al. 2022). The data presented in this paper aims to increase awareness of the metabolic profile of lithium to ensure safe and successful treatment with lithium, which is the mainstay of first-line treatment for bipolar disorder according to leading guidelines (Yatham et al. 2018, Malhi et al. 2023).

This study has some limitations: estimates based on naturalistic data from a pharmacovigilance program may suffer from inherent methodological drawbacks. Specifically, this type of analysis may suggest associations between drug and effect but cannot be used to prove causality. Furthermore, ADRs, such as weight gain in patients with a long outpatient medication history, could be underrepresented. This is known as reporting bias, where only extremely unusual cases of ADRs are reported by the responsible drug monitors in the collaborating hospitals. Further, the effect of reporting bias on the reporting of weight gain may be greater for drugs that have been used in psychiatry for a long time, such as lithium and valproate, than for newer drugs. It is also critical to note that a strict criterion of weight gain greater than 10% of the patient's initial weight was chosen. Lithium-induced weight gain may often be less pronounced, but may still affect the patient's adherence, quality of life, or health. Additionally, lithium-induced weight gain may develop gradually over a longer period of time (Solmi et al. 2020; Bowden et al. 2006) than with other drugs, such as SGAs, and therefore might stay undetected within our reporting system limited to the observation of hospitalized patients.

However, as already pointed out, our findings are consistent with methodically sound meta-analyses (Gomes-da-Costa et al. 2022; Kishi et al. 2022) suggesting that the risk of weight gain associated with lithium is lower than for other frequently prescribed mood stabilizers. This supports our view that the risk of weight gain related to lithium might have been overestimated in the past.

## Conclusions

Our analysis using data from a pharmacovigilance program, with lamotrigine as the reference drug, demonstrated an elevated occurrence of severe weight gain reports in patients treated with lithium; however, this increase was not statistically significant. In contrast, the use of olanzapine, quetiapine, or valproate was associated with a statistically significant higher risk of severe weight gain. Although lithium may perform better in this respect than some alternative compounds, careful monitoring of weight and metabolic parameters remains an important component of a safe therapy with lithium as with potential alternatives.

### Supplementary Information

**Additional file 1.** Reporting Odds Ratios (ROR) of severe weight gain compared to various reference drugs.

## Data Availability

The datasets used and/or analysed during the current study are available from the corresponding author on reasonable request.

## Abbreviations

ADR: Adverse drug reaction
AMSP: Arzneimittelsicherheit in der Psychiatrie
BMI: Body mass index
CI: Confidence interval
RCT: Randomized control study
ROR: Reported odds ratio
SWG: Severe weight gain
AEP: Antiepileptic drugs
FGA: First generation antipsychotics
NaSSA: Specific noradrenergic and specific serotonergic antidepressants
SGA: Second generation antipsychotics
SSRI: Selective serotonine reuptake inhibitors
SNRI: Selective serotonin–norepinephrine reuptake inhibitors
TCA: Tricyclic antidepressants
