# Supplementary material for: Controversies regarding lithium-associated weight gain: case–control study of real-world drug safety data
Source: Int J Bipolar Disord. 2023 Oct 15;11:34. doi: 10.1186/s40345-023-00313-8 (PMC10577117; doi:10.1186/s40345-023-00313-8)

**Additional Material**

**Controversies regarding Lithium-associated weight gain:**

**Case-control study of real-world drug safety data**

Waldemar Greil, Mateo de Bardeci, Bruno Müller-Oerlinghausen, Nadja Nievergelt, Hans Stassen, Gregor Hasler, Andreas Erfurth, Katja Cattapan, Eckart Rüther, Johanna Seifert, Sermin Toto, Stefan Bleich, Georgios Schoretsanitis

The analysis examined the reported occurrence of severe weight gain (SWG) between 2001-2015 from a total of 7,104 cases, focusing on 527 cases of SWG.

The figures (and tables) provided are present the Reporting Odds Ratio (ROR) for different medications compared to certain reference drugs. This ROR gives an indication of how likely a particular event (possibly an adverse drug reaction, ADR) is to occur with each drug in comparison to the reference drug. A ROR greater than 1 means the event is more likely with the compared drug than with the reference drug, while a ROR less than 1 means it is less likely.

Lithium, aripiprazole, carbamazepine, and high-potency FGAs (First-Generation Antipsychotics) were used as reference medications. The analysis provided ROR for each drug against the references, accompanied by confidence intervals and p-values obtained from the Chi-squared test.

We found that the order of drugs in terms of association with weight gain (SWG) remains the same for all drugs used as reference (order of SWG: from most to least):

1. Olanzapine
2. Quetiapine
3. Valproic acid
4. Lithium
5. Risperidone
6. Lamotrigine
7. Carbamazepine
8. Aripiprazole

The above order is consistent with the order of weight gain in Kishi et al 2022 in Molecular Psychiatry (Supplement, page 313), and the reported odds ratios are very similar. This indicates the validity of the data, in particular regarding lithium.

When comparing lithium with groups of psychotropic drugs such as ATYNL, SNRI, SSRI, NASSA, TZA, AEP, and TYNL_HP (explanation of the acronyms, see below), the order is:

1. ATYNL
2. SNRI
3. SSRI
4. NASSA
5. TZA
6. Lithium
7. AEP
8. TYNL_HP

When comparing TYNL_HP with ATYNL, SSRI, SNRI, NASSA, TZA, LI, and AEP, the order is:

1. ATYNL
2. SSRI
3. SNRI
4. NASSA
5. TZA
6. Lithium
7. AEP
8. TYNL_HP

Here are the key findings in detail:

- When Lithium (LI) is used as a reference (fig.1):
  1. Olanzapine (ROR: 7.198, p<0.001) and Quetiapine (ROR: 1.974, p=0.01) shows a significantly increased risk.
  2. Valproic acid, Risperidone, Lamotrigine, and Carbamazepine show no statistically significant increased or decreased risk compared to lithium.
  3. Aripiprazole shows a significantly decreased risk (ROR: 0.392, p<0.026).
- When carbamazepine is used as a reference (fig. 2):
  1. Olanzapine (ROR: 12.902, p<0.001), Quetiapine (ROR: 3.731, p=0.001), and Valproic acid (ROR: 3.208, p=0.002) have a significantly increased risk.
  2. Lithium (ROR: 2.247, p=0.06) and Risperidone (ROR: 2.14, p=0.06) show less pronounced increased risk but did not reach the significance level of 0.05.
  3. Lamotrigine and aripiprazole don't show significant differences.
- When aripiprazole is used as a reference (fig. 3):
  1. Olanzapine (ROR: 9.768, p<0.001), Quetiapine (ROR: 4.724, p<0.001), and Valproic acid (ROR: 3.446, p=0.001) show a significantly increased risk.
  2. Lithium (ROR: 2.553, p=0.026) and Risperidone (ROR: 2.238, p=0.033) also show an increased risk, but it is less pronounced.
  3. No significant difference is observed with Lamotrigine and Carbamazepine.
- When the group of FGAs high potency (TYNL_HP) is used as a reference (fig. 4):
  1. Olanzapine (ROR: 11.131, p<0.001), Quetiapine (ROR: 2.814, p<0.001), and Valproic acid (ROR: 2.638, p<0.001) show a significantly increased risk.
  2. Lithium (ROR: 1.818, p=0.013) and Risperidone (ROR: 1.672, p=0.008) also show increased risk but less pronounced.
  3. Lamotrigine, Carbamazepine, and Aripiprazole show no significant difference or decreased risk.
- When comparing lithium with groups of psychotropic drugs such as ATYNL, SNRI, SSRI, NASSA, TZA, AEP, and TYNL_HP (fig. 5):
  1. ATYNL (Second Generation Antipsychotics, SGAs) shows a significantly increased risk (ROR: 4.036, p=0.001).
  2. TYNL_HP (FGAs high potency, ROR: 0.55, p=0.013) shows a decreased risk.
  3. No significant difference is seen with other drug groups.
- When comparing TYNL_HP (FGAs high potency) with ATYNL, SSRI, SNRI, NASSA, TZA, LI, and AEP (fig. 6):
  1. ATYNL (SGA) has a significantly increased risk (ROR: 6.369, p=0).
  2. All other drugs show increased risk with similar magnitudes, and all are statistically significant.

In summary, olanzapine consistently showed a significant association with SWG across all reference medications. Lithium showed a significant association when compared with the following references: aripiprazole and FGAs high potency, and nearly significant when compared to carbamazepine.

In conclusion, the results with lithium as a reference are particularly clinically relevant. They provide an answer to the question of which drugs and drug groups are more favorable or less favorable (with regard to SWG). The evaluations show that, compared with lithium, aripiprazole and the group of high-potency FGAs are more favorable with regard to SWG. Olanzapine and quetiapine, as well as the group of SGAs, are more frequently associated with SWG than lithium. This may be relevant for patient education and shared decision-making in psychotropic drug treatment.

**Legend:**

The database is originally in German; therefore, some acronyms might resemble the German language. Here is the explanation of the acronyms:

- AEP: Antiepileptic drugs
- ATYNL: Second-generation antipsychotics (SGA).
- LI: Lithium
- NASSA: noradrenergic and specific serotonergic antidepressants
- SSRI: Selective Serotonin Reuptake Inhibitors.
- SNRI: Serotonin and Norepinephrine Reuptake Inhibitors.
- TYNL_HP: First-generation antipsychotics (FGA) with high potency
- TZA: Tricyclic Antidepressants.

**References:**

Kishi T, Ikuta T, Matsuda Y, Sakuma K, Okuya M, Nomura I, Hatano M, Iwata N. Pharmacological treatment for bipolar mania: a systematic review and network meta-analysis of double-blind randomized controlled trials. Mol Psychiatry. 2022;27:1136-44.

Figure S1

Reference: LI (lithium)

| **ROR** | **LI** | **N** | **confint_lower** | **confint_higher** | **p_val_chi** | **label** | **order** |
| --- | --- | --- | --- | --- | --- | --- | --- |
| 7.198 | 31 | 247 | 4.278 | 12.109 | <0.001 | Olanzapine | 8.00 |
| 1.974 | 31 | 104 | 1.188 | 3.278 | 0.01 | Quetiapine | 7.00 |
| 1.434 | 31 | 61 | 0.893 | 2.304 | 0.167 | Valproic acid | 6.00 |
| 1 | - | - | 0 | 0 | - | LI | 5.00 |
| 0.907 | 31 | 60 | 0.579 | 1.42 | 0.755 | Risperidone | 4.00 |
| 0.476 | 31 | 8 | 0.195 | 1.164 | 0.141 | Lamotrigine | 3.00 |
| 0.445 | 31 | 9 | 0.201 | 0.984 | 0.061 | Carbamazepine | 2.00 |
| 0.392 | 31 | 9 | 0.177 | 0.865 | 0.026 | Aripiprazole | 1.00 |


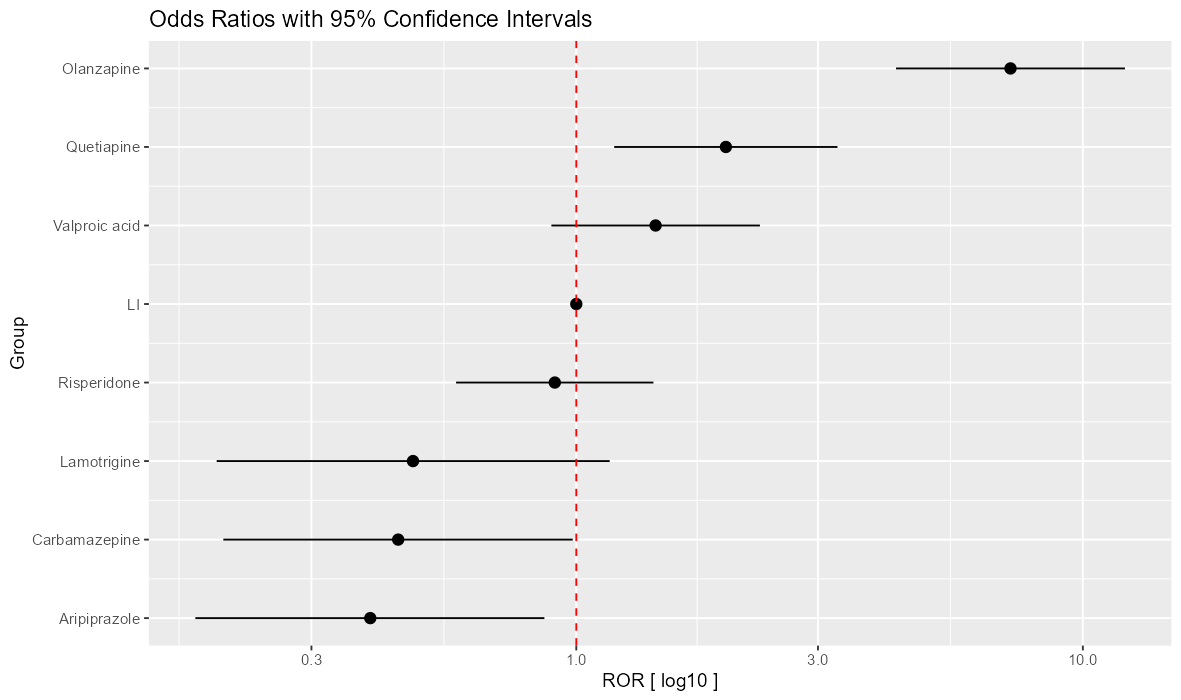


Figure S2

Reference: carbamazepine

| **ROR** | **Carbamazepine** | **N** | **confint_lower** | **confint_higher** | **p_val_chi** | **label** | **order** |
| --- | --- | --- | --- | --- | --- | --- | --- |
| 12.902 | 9 | 247 | 5.677 | 29.321 | <0.001 | Olanzapine | 8.00 |
| 3.731 | 9 | 104 | 1.714 | 8.12 | 0.001 | Quetiapine | 7.00 |
| 3.208 | 9 | 61 | 1.515 | 6.793 | 0.002 | Valproic acid | 6.00 |
| 2.247 | 9 | 31 | 1.016 | 4.969 | 0.061 | LI | 5.00 |
| 2.14 | 9 | 60 | 1.01 | 4.533 | 0.06 | Risperidone | 4.00 |
| 1.164 | 9 | 8 | 0.442 | 3.065 | 0.953 | Lamotrigine | 3.00 |
| 1 | - | - | 0 | 0 | - | Carbamazepine | 2.00 |
| 0.884 | 9 | 9 | 0.346 | 2.255 | 0.984 | Aripiprazole | 1.00 |


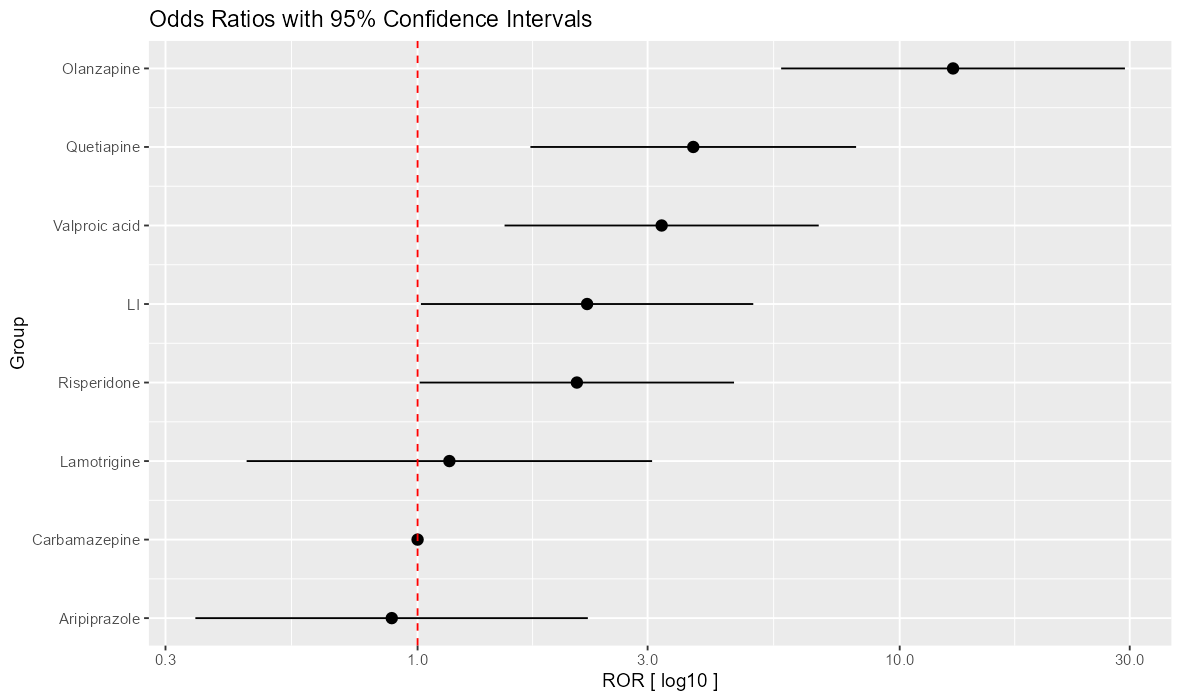


Fig. S3

Reference: aripiprazole

| **ROR** | **Aripiprazole** | **N** | **confint_lower** | **confint_higher** | **p_val_chi** | **label** | **order** |
| --- | --- | --- | --- | --- | --- | --- | --- |
| 9.768 | 9 | 247 | 4.961 | 19.232 | <0.001 | Olanzapine | 8.00 |
| 4.724 | 9 | 104 | 2.052 | 10.877 | <0.001 | Quetiapine | 7.00 |
| 3.446 | 9 | 61 | 1.627 | 7.296 | 0.001 | Valproic acid | 6.00 |
| 2.553 | 9 | 31 | 1.156 | 5.64 | 0.026 | LI | 5.00 |
| 2.238 | 9 | 60 | 1.098 | 4.561 | 0.033 | Risperidone | 4.00 |
| 1.319 | 9 | 8 | 0.471 | 3.69 | 0.794 | Lamotrigine | 3.00 |
| 1.132 | 9 | 9 | 0.443 | 2.889 | 0.984 | Carbamazepine | 2.00 |
| 1 | - | - | 0 | 0 | - | Aripiprazole | 1.00 |


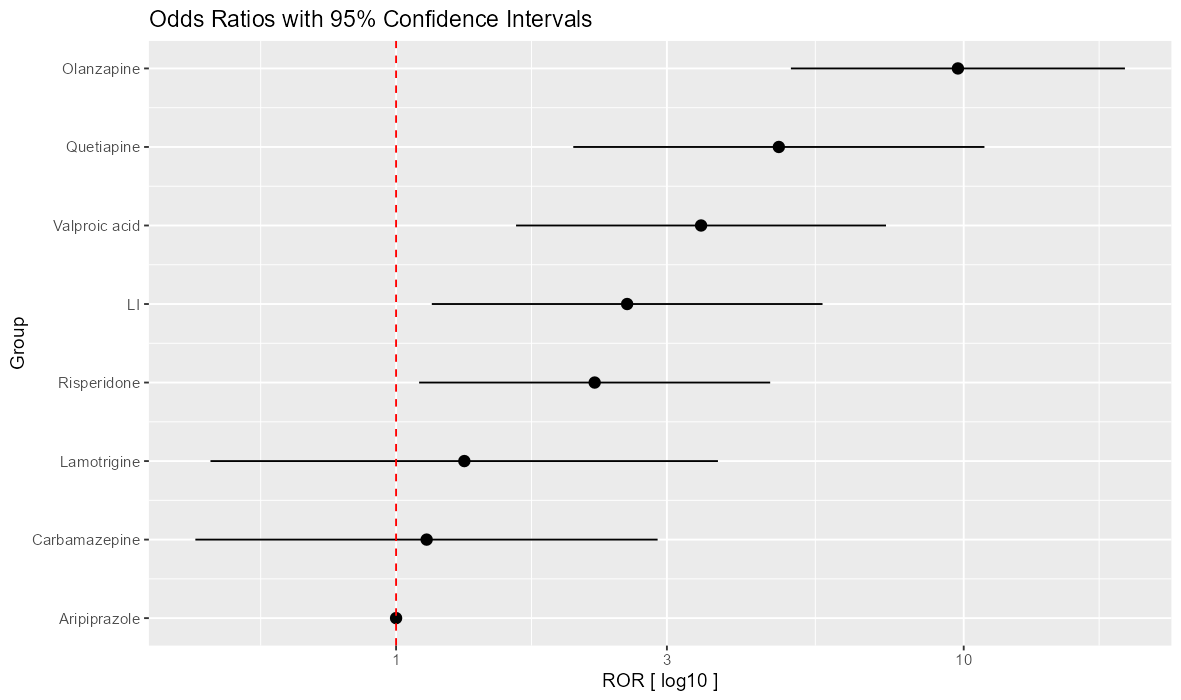


Figure S4

Reference: TYNL_HP / FGA high potency

| **ROR** | **TYNL_HP** | **1** | **confint_lower** | **confint_higher** | **p_val_chi** | **label** | **order** |
| --- | --- | --- | --- | --- | --- | --- | --- |
| 11.131 | 66 | 247 | 7.83 | 15.823 | <0.001 | Olanzapine | 9.00 |
| 2.814 | 66 | 104 | 1.995 | 3.97 | <0.001 | Quetiapine | 8.00 |
| 2.638 | 66 | 61 | 1.799 | 3.869 | <0.001 | Valproic acid | 7.00 |
| 1.818 | 66 | 31 | 1.156 | 2.86 | 0.013 | LI | 6.00 |
| 1.672 | 66 | 60 | 1.157 | 2.418 | 0.008 | Risperidone | 5.00 |
| 1 | - | - | 0 | 0 | - | TYNL_HP | 4.00 |
| 0.925 | 66 | 8 | 0.438 | 1.953 | 0.984 | Lamotrigine | 3.00 |
| 0.636 | 66 | 9 | 0.272 | 1.486 | 0.384 | Carbamazepine | 2.00 |
| 0.607 | 66 | 9 | 0.275 | 1.337 | 0.279 | Aripiprazole | 1.00 |


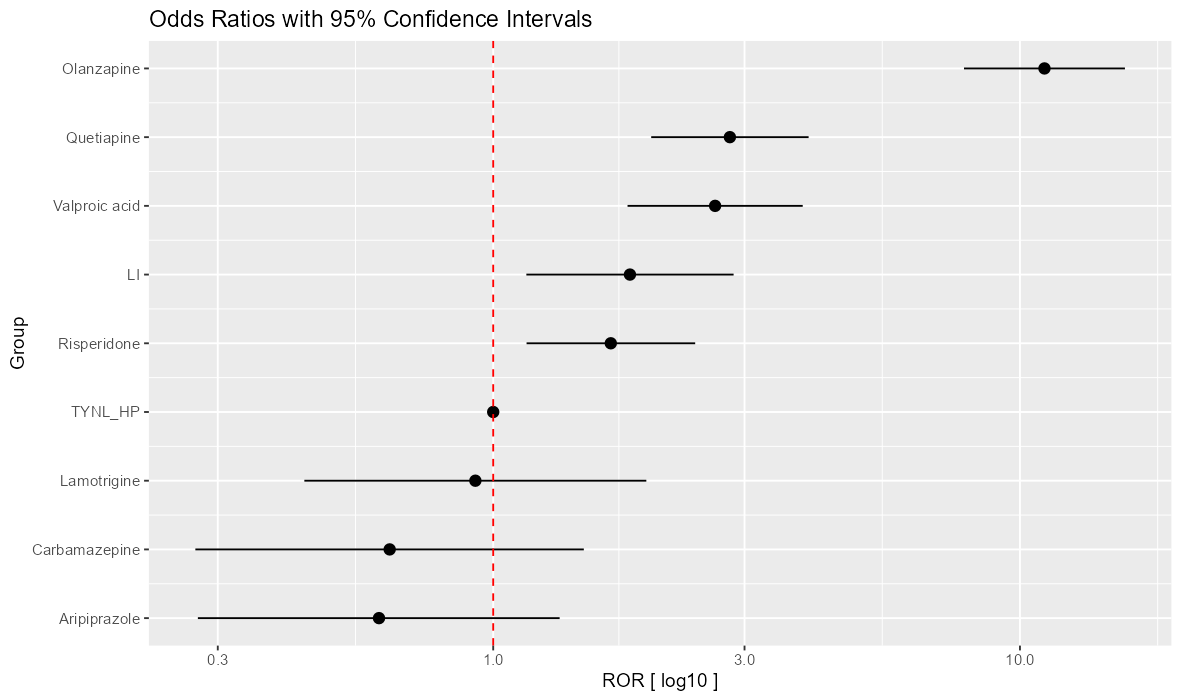


Figure S5

Reference: LI (lithium)

| **ROR** | **LI** | **N** | **confint_lower** | **confint_higher** | **p_val_chi** | **label** | **order** |
| --- | --- | --- | --- | --- | --- | --- | --- |
| 4.036 | 31 | 449 | 1.65 | 9.874 | 0.001 | ATYNL | 8.00 |
| 1.322 | 31 | 113 | 0.818 | 2.138 | 0.305 | SNRI | 7.00 |
| 1.27 | 31 | 75 | 0.821 | 1.963 | 0.331 | SSRI | 6.00 |
| 1.2 | 31 | 75 | 0.769 | 1.873 | 0.488 | NASSA | 5.00 |
| 1.112 | 31 | 39 | 0.682 | 1.814 | 0.764 | TZA | 4.00 |
| 1 | 31 | 103 | 0 | 0 | - | LI | 3.00 |
| 0.955 | - | - | 0.601 | 1.517 | 0.94 | AEP | 2.00 |
| 0.55 | 31 | 66 | 0.35 | 0.865 | 0.013 | TYNL_HP | 1.00 |


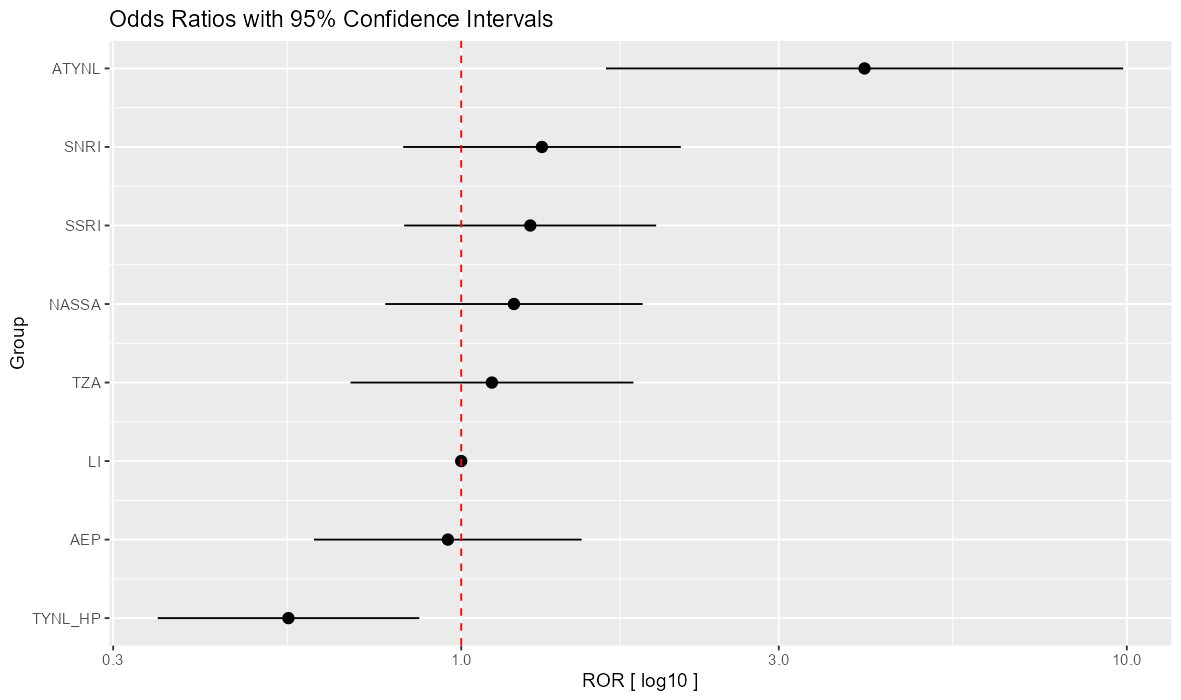


Figure S6

Reference: TYNL_HP (FGA high potency)

| **ROR** | **TYNL_HP** | **N** | **confint_lower** | **confint_higher** | **p_val_chi** | **label** | **order** |
| --- | --- | --- | --- | --- | --- | --- | --- |
| 6.369 | 66 | 449 | 3.78 | 10.731 | <0.001 | ATYNL | 8.00 |
| 2.2 | 66 | 113 | 1.582 | 3.059 | <0.001 | SSRI | 7.00 |
| 2.119 | 66 | 75 | 1.484 | 3.026 | <0.001 | SNRI | 6.00 |
| 2.068 | 66 | 75 | 1.452 | 2.945 | <0.001 | NASSA | 5.00 |
| 1.871 | 66 | 39 | 1.215 | 2.883 | 0.006 | TZA | 4.00 |
| 1.818 | 66 | 103 | 1.156 | 2.86 | 0.013 | LI | 3.00 |
| 1.789 | 66 | 31 | 1.263 | 2.535 | 0.001 | AEP | 2.00 |
| 1 | - | - | 0 | 0 | - | TYNL_HP | 1.00 |


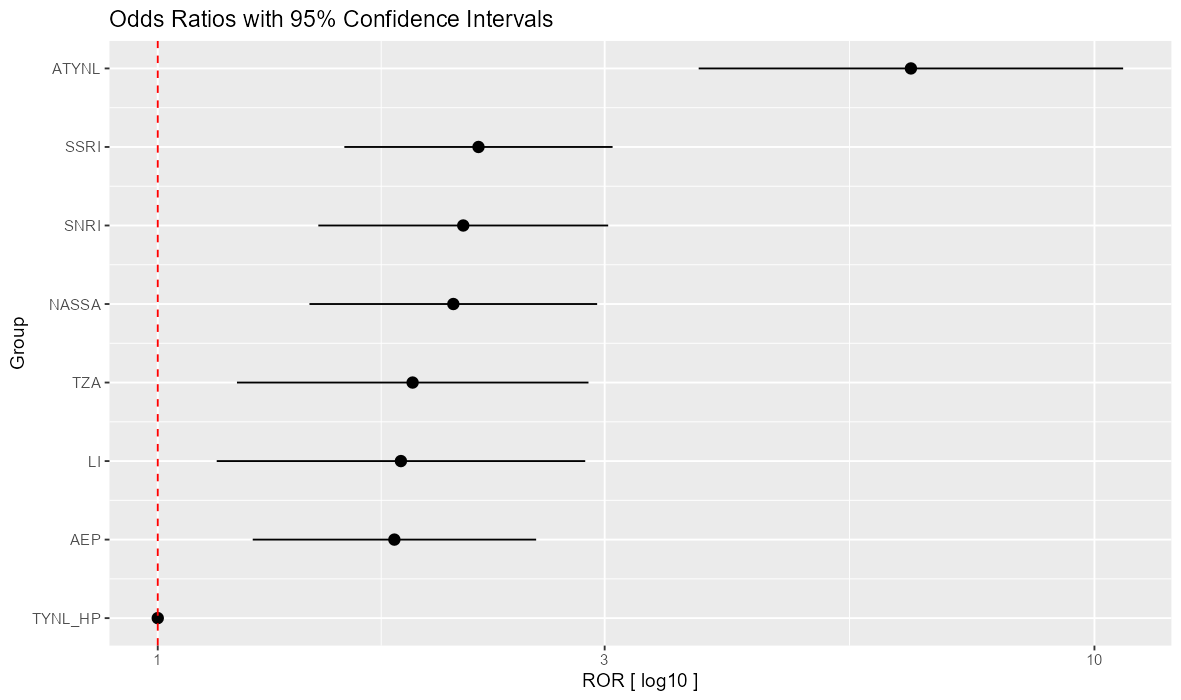

Supplement: Supplementary file 1 — Additional file 1. Reporting Odds Ratios (ROR) of severe weight gain compared to various reference drugs. [file 40345_2023_313_MOESM1_ESM.docx]
